# Characterization of Novel Plantaricin-Derived Antiviral Peptides Against Flaviviruses

**DOI:** 10.3390/ijms26031038

**Published:** 2025-01-25

**Authors:** Abubakr A. M. Omer, Sanjiv Kumar, Robert Selegård, Torbjörn Bengtsson, Hazem Khalaf

**Affiliations:** 1 School of Medical Sciences, Faculty of Medicine and Health, Örebro University, 701 82 Örebro, Sweden; abubakr.omer@oru.se (A.A.M.O.); drsanjivk@gmail.com (S.K.); torbjorn.bengtsson@oru.se (T.B.); 2 Division of Biophysics and Bioengineering, Department of Physics, Chemistry and Biology, Linköping University, 581 83 Linköping, Sweden; robert.selegard@icloud.com

**Keywords:** antiviral, plantaricin, flaviviruses, West Nile virus, zika virus, molecular docking

## Abstract

Flaviviruses, including West Nile virus, Zika virus, and Dengue virus, pose global health challenges due to their distribution, pathogenicity, and lack of effective treatments or vaccines. This study investigated the antiviral activity of novel truncated peptides derived from the two-peptide plantaricins PLNC8 αβ, PlnEF, PlnJK, and PlnA. The antiviral potential was predicted using machine learning tools, followed by in vitro evaluation against the Kunjin virus using plaque reduction assays in Vero cells. Molecular docking assessed peptide interactions with KUNV and ZIKV. Full-length and truncated peptides from PlnA, PlnE, PlnF, PlnJ, and PlnK demonstrated limited antiviral efficacy against KUNV in vitro, despite in silico predictions suggesting antiviral potential for PlnA, PlnE, and PlnJ. Large discrepancies were observed between the predicted and experimentally determined activities. However, complementary two-peptide plantaricins PlnEF and PlnJK exhibited significant synergistic effects. Furthermore, the truncated peptides PLNC8 α1-15 and PLNC8 β1-20 reduced KUNV viral load by over 90%, outperforming their full-length counterparts. Molecular docking revealed interactions of PLNC8 α and PLNC8 β, and their truncated variants, with KUNV and ZIKV, suggesting a mechanism involving viral envelope disruption. These findings highlight the potential of plantaricin-derived peptides as promising antiviral candidates against flaviviruses, warranting further investigation into their mechanisms and applications.

## 1. Introduction

Flaviviruses are a group of enveloped, positive-sense, single-stranded RNA viruses belonging to the *Flaviviridae* family, primarily transmitted by arthropod vectors, such as mosquitoes and ticks [1,2]. This group includes globally recognized pathogens, such as Dengue virus (DENV), Zika virus (ZIKV), West Nile virus (WNV), Kunjin virus (KUNV), Yellow fever virus (YFV), Japanese encephalitis virus (JEV), and tick-borne encephalitis virus (TBEV) [3,4,5]. Flaviviruses possess a lipid bilayer envelope derived from the host endoplasmic reticulum (ER), which plays a critical role in viral entry and assembly [6]. They are distributed worldwide and can cause a range of diseases, from mild febrile illness to severe conditions, such as hemorrhagic fever, encephalitis, and congenital anomalies, with Zika virus being notably linked to microcephaly [7,8]. The development of novel antiviral agents is crucial due to the lack of specific antiviral therapies and effective vaccines for several flaviviruses, including ZIKV and WNV [9,10,11].

Antimicrobial peptides (AMPs) are naturally occurring small molecules acting against diverse pathogens, including bacteria, viruses, fungi, and parasites [12,13,14,15]. AMPs are generally characterized by their amphipathic structures, which allow them to selectively interact with microbial membranes and cause membrane disruption, pore formation, and, ultimately, microbial cell death [16,17,18]. In addition to their direct antimicrobial effects, certain AMPs possess notable immunomodulatory properties, such as defensins and cathelicidins, which enhance the immune responses of the host against invading pathogens [19,20,21]. For instance, LL-37, which is composed of the C-terminal part of human cathelicidin, is known to modulate inflammation by influencing cytokine production and chemotaxis [22], while defensins help recruit immune cells to the site of infection and promote wound healing [23]. These immunomodulatory effects, in combination with their broad-spectrum antimicrobial properties and relatively low risk of resistance, make AMPs interesting candidates for research in developing new antiviral approaches [24,25,26,27,28].

Among AMPs, two-peptide bacteriocins (Class IIb bacteriocins) are notable for their dual-component structure, requiring two complementary peptides to exert full antimicrobial activity. These AMPs are produced by lactic acid bacteria, such as *Lactobacillus plantarum*, including plantaricin (Pln) EF, JK, and NC8 αβ, which exhibited potent antimicrobial activity against a variety of pathogens, including bacteria, fungi, and viruses [29,30,31,32]. The synergistic two-peptide system enables these bacteriocins to form transmembrane pores in bacterial membranes, resulting in cell death through the leakage of essential cellular contents [33]. Interestingly, in *Lactobacillus plantarum* C11, the production of two-peptide bacteriocins, such as PlnEF and PlnJK, is regulated by PlnA, which is a peptide pheromone that triggers bacteriocin synthesis in response to environmental signals. While PlnA triggers the synthesis and release of these potent bacteriocins, its actual antimicrobial effect is believed to be much lower than that caused by PlnEF, PlnJK, and PLNC8 αβ [29,33,34].

Two-peptide bacteriocins, including PlnEF, PlnJK, and PLNC8 αβ, have previously been highlighted for their strong antibacterial activity and low cytotoxicity toward host cells [35,36]. In previous studies, we have demonstrated the potent and rapid antiviral activity of PLNC8 αβ against enveloped viruses, hypothesizing that it disrupts viral lipid membranes and thereby inhibits viral entry and replication [37]. Beyond PLNC8 αβ, the antiviral activity of other plantaricins (PlnA, PlnEF, and PlnJK) against enveloped viruses has been less explored. Consequently, further research is needed to elucidate these peptides’ mechanisms of action and therapeutic potential against viral infections. This study focuses on characterizing the antiviral activities of full-length peptides, including PLNC8 α, PLNC8 β, Pln A, E, F, J, and K, and novel truncated peptides derived from their respective sequences. The exploration of truncated variants was based on the hypothesis that shorter peptides may adopt more favorable conformations, exposing key functional residues to improve interactions with viral components. Supporting this approach, previous studies have demonstrated that truncation can enhance biological activity by removing redundant regions while preserving or amplifying critical functional motifs [38,39,40,41]. By integrating computational and experimental methodologies, this study aims to support the rational design and optimization of peptides with enhanced antiviral activities and therapeutic potential.

## 2. Results

### 2.1. Prediction and Determination of Antiviral Activity

Advanced computational tools were initially employed to assess the antiviral potential of full-length plantaricins, PLNC8 α, PLNC8 β, PlnA, E, F, J, and K, and their respective truncated peptides. The antiviral activity was systematically evaluated using two different available prediction tools, FIRM-AVP and AVPpred. Analyses of different truncated variants of plantaricins were included with the aim of investigating whether the sequences of full-length peptides could be optimized for their antiviral efficacy. All the peptides were subjected to rigorous in vitro testing to assess their antiviral activities against the flavivirus KUNV, performed by using plaque reduction assays. All full-length Pln peptides, except PlnK, were predicted to have antiviral properties; however, their activity against KUNV did not exceed 30% reduction in viral load (Table 1).

**Figure 1:**
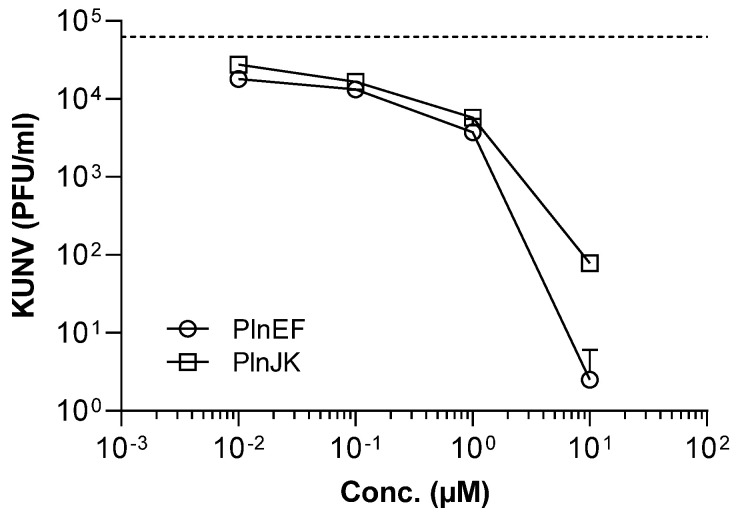
Antiviral activity of the two-peptide plantaricins EF and JK against KUNV. The flavivirus Kunjin (KUNV, 1 × 10^5^) was suspended in a cell culture medium (DMEM) and either left untreated or exposed to different concentrations of PlnEF, or PlnJK for 1 h. The suspensions were then added to a semi-confluent layer of Vero cells for 1 h, followed by the addition of overlay media and incubation for 3 days. Viral load was quantified by performing the plaque assay. The dotted line shows the number of KUNV (PFU/mL) in the untreated positive control. Results are presented as the mean with standard deviation of two independent experiments, each in duplicate. The two-peptide bacteriocins PlnEF and PlnJK are efficient against KUNV and can substantially reduce the viral load.

Selected Pln-derived truncated variants were also tested, and their antiviral activities were not improved compared to their parent full-length peptides. Two-peptide bacteriocins, including PlnEF and PlnJK, are known to exert full antimicrobial activity based on the two complementary peptides. The antibacterial effects of these bacteriocins have previously been demonstrated, particularly against Gram-positive bacteria, which encouraged us to determine their antiviral activity against KUNV. The combinations PlnEF and PlnJK demonstrated a substantial and dose-dependent antiviral activity against KUNV by significantly reducing the viral load compared to their individual counterparts (Figure 1). The highest concentration of both PlnEF and PlnJK, 10 µM, caused > 99.9% reduction in viral load. These results show the synergistic interactions between the peptides, i.e., PlnE/PlnF and PlnJ/PlnK, that enhance their overall antiviral activity and indicate their broad spectrum of activity by targeting viral envelopes.

Furthermore, although full-length PLNC8 α was predicted to possess antiviral properties, the in vitro experiments showed ~35% reduction in viral load (Table 2). In contrast, the truncated peptide PLNC8 α1-15 showed >90% reduction in KUNV, surpassing the antiviral activity of its parent full-length peptide, although both prediction tools failed to identify its antiviral properties. The remaining truncated peptides showed varied predicted antiviral activities; however, no variant exceeded the observed activity of full-length PLNC8 α.

The prediction of the antiviral properties of full-length PLNC8 β corresponded well with its determined activity against KUNV (Table 3). Among the different truncated peptides, PLNC8 β1-20 showed the most potent antiviral activity against KUNV, which was predicted by FIRM-AVP, but not AVPpred, to possess antiviral properties.

Interestingly, large discrepancies were observed when comparing the scores from the prediction tools to the experimentally determined antiviral activity of the different peptides using plaque reduction assays. Several peptides with high predicted scores showed moderate or low efficacy in the plaque reduction assays, while some truncated peptides with modest predicted scores exhibited strong antiviral effects.

### 2.2. Molecular Analysis of Peptide Properties and Interactions with Viral Proteins

**Figure 2:**
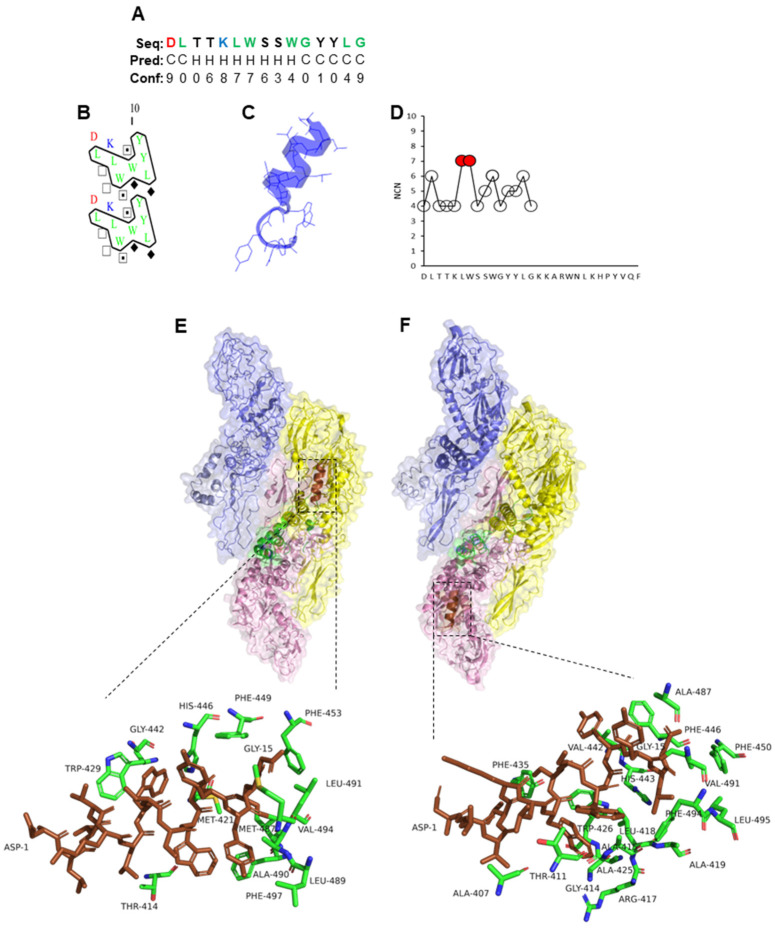
Properties of PLNC8 α1-15 and its interaction with flaviviruses. (**A**) Amino acid sequence (1D) and prediction of α-helices (Hs) and coils (Cs). The colors green, red, blue, and black represent amino acids that are hydrophobic, acidic, basic, and neutral, respectively. (**B**) Two-dimensional representation in which horizontal clusters of hydrophobic amino acids predict the formation of α-helices. Green and blue colors indicate hydrophobic and basic residues, respectively. (**C**) Predicted 3D structure of PLNC8 α1-15. (**D**) The number of contact neighbors (NCNs). MIRs (red) were defined based on the thresholds > 6 and show that the residues L and W are important for peptide folding. (**E**) Predicted interaction of PLNC8 α1-15 with structural E and M proteins of Zika virus (strain H/PF/2013, pdb:5IZ7, [42]) and (**F**) West Nile virus (Kunjin strain, pdb:7KVA, [43]). PLNC8 α1-15 shows interactions with the stems and transmembrane regions of Zika and Kunjin.

The truncated peptide variants PLNC8 α1-15 and PLNC8 β1-20 showed promising antiviral activity that encouraged us to elucidate their underlying interaction mechanisms with flavivirus proteins, compared to their corresponding full-length peptides. Furthermore, in silico molecular analyses were conducted to characterize their properties based on their amino acid sequences. The secondary structure prediction of PLNC8 α1-15 shows that the peptide has a tendency towards adopting an α-helix when analyzed and projected in 1D, 2D, and 3D (Figure 2A–C). The amino acids leucine (L) and tryptophan (W) were identified to be the most interacting residues (MIRs) within the sequence (Figure 2D), and thus, important for peptide folding. PLNC8 α1-15 interacted with ZIKV (Figure 2E) and KUNV (Figure 2F) with motifs at the stem and transmembrane regions, suggesting possible access and binding to phospholipids in the viral envelope.

**Figure 3:**
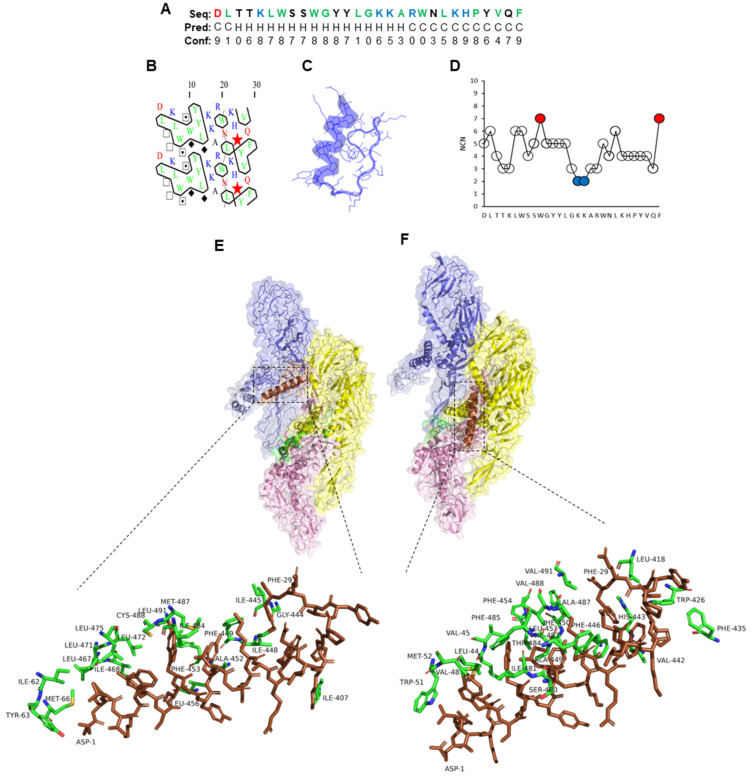
Properties of full-length PLNC8 α and its interaction with flaviviruses. (**A**) Amino acid sequence (1D) and prediction of α-helices (Hs) and coils (Cs). The colors green, red, blue, and black represent amino acids that are hydrophobic, acidic, basic, and neutral, respectively. (**B**) Two-dimensional representation in which horizontal clusters of hydrophobic amino acids predict the formation of α-helices. Green and blue colors indicate hydrophobic and basic residues, respectively. (**C**) Predicted 3D structure of PLNC8 α. (**D**) The number of contact neighbors (NCNs), with MIRs (red) and LIRs (blue) of PLNC8 α were defined based on the thresholds > 6 and ≤3, respectively, and show that the MIR residues W and F are important for peptide folding. Interaction sites between the peptide and viral proteins were performed using FroDock. (**E**) Predicted interaction of PLNC8 α with structural E and M proteins of Zika virus (strain H/PF/2013, pdb:5IZ7) and (**F**) West Nile virus (Kunjin strain, pdb:7KVA). PLNC8 α interacts primarily with the stems and transmembrane regions of Zika and Kunjin.

In contrast, the N-terminal region of full-length PLNC8 α (29 amino acid peptide) was predicted to form an α-helix, while the C-terminal region formed random coils (Figure 3A–C). The hydrophobic amino acids tryptophan (W) and phenylalanine (F) were identified as MIRs and are most probably important contributors to peptide stability and folding, while two repeated lysine (K) were least interacting residues (LIRs), suggesting an important function in peptide activity (Figure 3D). PLNC8 α interacted mainly with the stems and transmembrane regions of ZIKV (Figure 3E) and KUNV (Figure 3F).

**Figure 4:**
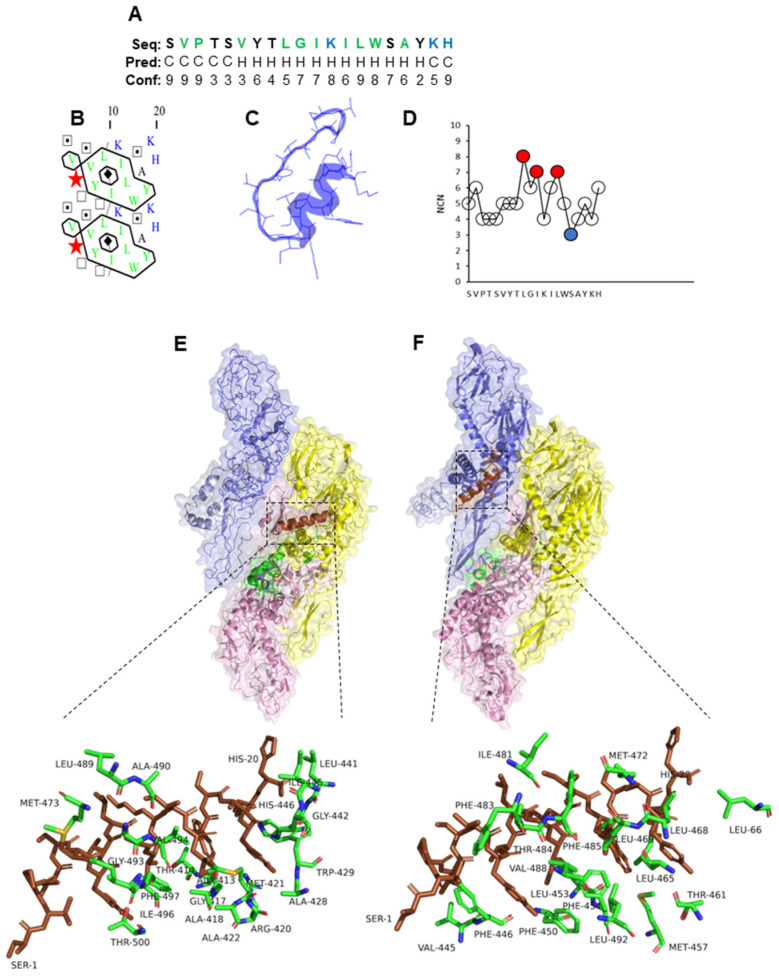
Properties of PLNC8 β1-20 and its interaction with flaviviruses. (**A**) Amino acid sequence (1D) and prediction of α-helices (Hs) and coils (Cs). The colors green, blue, and black represent amino acids that are hydrophobic, basic, and neutral, respectively. (**B**) Two-dimensional representation in which horizontal clusters of hydrophobic residues predict the formation of α-helices. Green and blue colors indicate hydrophobic and basic residues, respectively. (**C**) Predicted 3D structure of PLNC8 β1-20. (**D**) The number of contact neighbors (NCNs), with MIRs (red) and LIRs (blue) of PLNC8 β1-20 that were defined based on the thresholds > 6 and ≤3, respectively, show that the MIR hydrophobic residues L, I, and L are important for folding. (**E**) Predicted interaction of PLNC8 β1-20 with structural E and M proteins of Zika virus (strain H/PF/2013, pdb:5IZ7) and (**F**) West Nile virus (Kunjin strain, pdb:7KVA). PLNC8 β1-20 interacts with amino acid residues in Zika and Kunjin that are part of the stem and transmembrane regions.

Furthermore, the secondary structure analysis of PLNC8 β1-20 showed a tendency towards random coil formation at the N-terminal region, while the C-terminal region adopted an α-helix (Figure 4A–C). Two leucine (L) and one isoleucine (I) residues were identified as MIRs and, thus, play crucial roles in maintaining structural integrity, and serine (S) was an LIR within the amino acid sequence (Figure 4D). The molecular docking of PLNC8 β1-20 with ZIKV and KUNV demonstrated strong interactions with the transmembrane regions of the structural viral proteins (Figure 4E,F).

**Figure 5:**
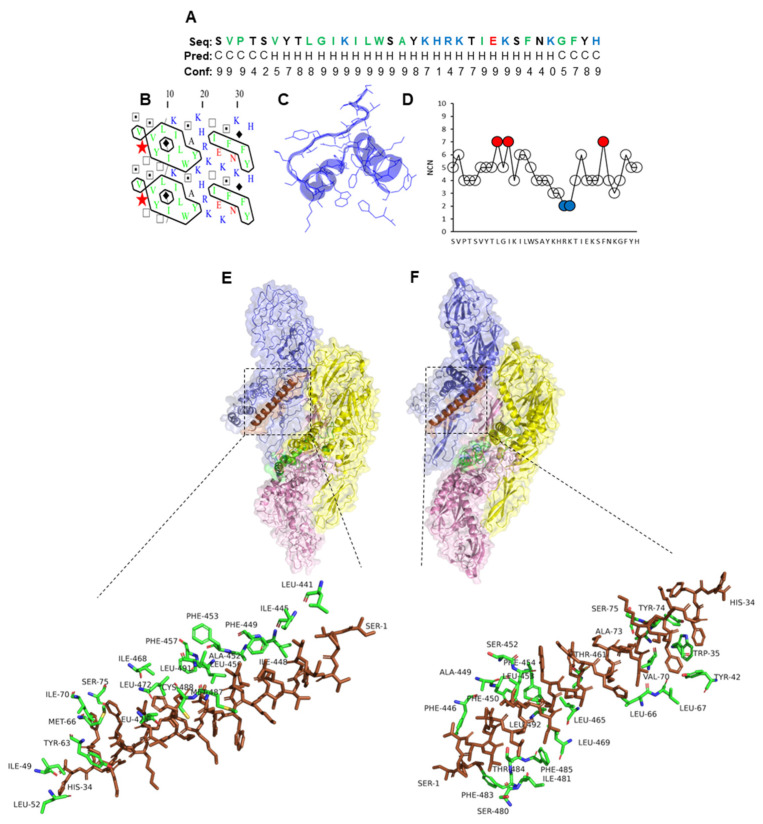
Properties of full-length PLNC8 β and its interaction with flaviviruses. (**A**) Amino acid sequence (1D) and prediction of α-helices (Hs) and coils (Cs). The colors green, red, blue, and black represent amino acids that are hydrophobic, acidic, basic, and neutral, respectively. (**B**) Two-dimensional representation in which horizontal clusters of hydrophobic amino acids predict the formation of α-helices. Green and blue colors indicate hydrophobic and basic residues, respectively. (**C**) Predicted 3D structure of PLNC8 β. (**D**) Number of contact neighbors (NCNs), with MIRs (red) and LIRs (blue) of PLNC8 β that were defined based on the thresholds > 6 and ≤3, respectively, show that the MIR hydrophobic residues L, I, and F are important for peptide folding. (**E**) Predicted interaction of PLNC8 β with structural E and M proteins of Zika virus (strain H/PF/2013, pdb:5IZ7) and (**F**) West Nile virus (Kunjin strain, pdb:7KVA).

A full-length PLNC8 β, composed of 34 residues, was predicted to be composed of a random coil at the N-terminal region and an α-helix region at the C-terminal region (Figure 5A–C). Similarly, the MIRs within the sequence were identified as L, I, and F, which are residues with hydrophobic side chains, while the LIRs were arginine (R) and lysine (K), amino acids with cationic side chains (Figure 5D). PLNC8 β showed strong interactions with the transmembrane regions (M protein). PLNC8 β displayed interactions with the structural E proteins in both ZIKV (Figure 5E) and KUNV (Figure 5F). However, the interaction with the stems and transmembrane regions (M protein) is stronger as it includes interaction with additional residues, suggesting access and potentially binding to phospholipids in the viral envelope. A summary of all the interaction energies of full-length and truncated peptides with KUNV and ZIKV is presented in Table 4.

## 3. Discussion

There is an urgent need for new antiviral treatments to combat the global health threats caused by severe flavivirus infections against which specific licensed therapies and effective vaccines are lacking in many cases [9,10,11]. This study aimed to explore the antiviral efficacy of various full-length and truncated peptides of plantaricins against KUNV, by combining in silico predictions with in vitro validation. Our findings underscore the value of integrating computational predictions with experimental assays in identifying effective antiviral candidates. In particular, the truncated peptides PLNC8 α1-15 and PLNC8 β1-20 demonstrated remarkable antiviral efficacy, achieving over 90% viral load reduction as revealed in the plaque reduction assays, surpassing the activity of their full-length counterparts. This enhanced antiviral potency observed in the truncated peptides suggests that specific peptide fragments may adopt a more favorable structural conformation, improving their ability to interact with and disrupt viral components effectively. Shorter peptide variants may expose key functional residues more efficiently, enhancing their interaction with viral membranes or proteins. This observation aligns with previous studies demonstrating that truncated peptides can retain or even surpass the biological activity of their full-length counterparts, potentially due to the removal of structurally redundant regions while preserving or amplifying critical functional motifs [38,39,40,41]. Future research will focus on a more detailed characterization of the active domains responsible for antiviral activity, as well as exploring the efficacy of various truncated peptide formulations, which could lead to the identification of optimized peptide candidates for therapeutic development.

FIRM-AVP and AVPpred, which were used in this study, are computational tools designed to predict antiviral peptides (AVPs) using distinct methodologies. FIRM-AVP employs machine learning to analyze both primary and secondary structural features, such as hydrophobicity, charge, and structural motifs, ranking their importance for antiviral activity [44]. In contrast, AVPpred relies on a support vector machine (SVM)-based approach, focusing on sequence-derived features like amino acid composition and charge, with predictions expressed as probability scores for activity [45]. While FIRM-AVP incorporates structural complexity, AVPpred provides a simpler framework but lacks secondary structure analysis [44]. Our study evaluated the predicted antiviral activity by the FIRM-AVP and AVPpred software (version 1) for full-length and truncated versions of PLNC8 α and PLNC8 β, respectively, along with other plantaricins. Predictions were corroborated by in vitro assays. This approach is generally recommended to streamline the identification of promising antiviral candidates and save time and resources by focusing on peptides with high predicted antimicrobial efficacy [46,47]. However, in our study, we observed discrepancies between the predicted antiviral scores and actual in vitro activity against KUNV. Several peptides with high predicted scores showed moderate or limited efficacy in the plaque reduction assays, while some truncated peptides with modest predicted scores exhibited potent antiviral effects, including PLNC8 α1-15 and PLNC8 β1-20. These discrepancies likely reflect the complexity of antiviral peptide behavior in biological systems, where factors such as peptide stability, folding, target accessibility, and interaction with the viral envelope play crucial roles that may not be fully captured by the used predictive models. This highlights the necessity of empirical validation to confirm antiviral potency and suggest areas where computational tools could be refined for greater predictive accuracy in future studies. Most of the currently available machine learning models that are used to predict the activity of AMPs in general often lack sufficient negative and positive controls and aggregate data from heterogeneous sources with varying experimental conditions. This inconsistency undermines the generalizability of the predictions, as some peptides may only demonstrate activity against specific viral or bacterial strains [48]. Addressing these issues through standardized datasets and advanced AI-based tools could enhance the accuracy of computational predictions and their future applicability [47,49].

The observation that a combination of peptides such as PlnEF and PlnJK exhibited strong antiviral effects at low micromolar and even sub-micromolar concentrations is particularly noteworthy. This confirms the potent synergistic interactions between the two individual peptides in two-peptide bacteriocins, which correlates to their established synergy in antibacterial applications. Indeed, previous studies on the antibacterial effects of PlnE and PlnF have demonstrated that the combination of these two peptides surpasses the effects of each peptide alone. This is likely due to their capacity to form stable and cooperative pore structures within bacterial membranes, which are associated with their structural motifs like GxxxG and GxxxG-like sequences that enable transmembrane helix–helix formation. These motifs enhance the stability and efficacy of peptide complexes, leading to a more effective disruption of bacterial membranes, compared to the ability of individual peptides [50,51]. Our previous research has demonstrated that PLNC8 αβ, which is a two-peptide bacteriocin that belongs to the same group as that of PlnEF and PlnJK, shows potent antibacterial and antiviral efficacy [36,37], which may point to a broader mechanism of action within two-peptide bacteriocins. We hypothesize that combinations such as PlnEF, PlnJK, and PLNC8 αβ may destabilize viral envelopes through cooperative membrane disruption, akin to their effects on bacterial membranes. In this mechanism, one peptide may initiate partial destabilization, while the second amplifies the effect, resulting in greater viral inactivation. This cooperative action could significantly impair viral entry and replication, providing a robust antiviral strategy.

In silico analyses and molecular docking provided key insights into the mechanism of action of these peptides. Docking studies revealed strong interactions between PLNC8 α, PLNC8 β, and their truncated versions (PLNC8 α1-15 and PLNC8 β1-20) with the E and M proteins of KUNV and ZIKV, critical components of the viral envelope. The low binding energies and predicted interaction sites suggest that these peptides disrupt viral envelope integrity by destabilizing key structural proteins, likely reducing extracellular virions and limiting viral spread.

The reliability of the docking findings is supported using FRODOCK, a robust docking platform known for its high accuracy in protein–peptide interactions. Benchmarking studies have demonstrated its effectiveness in modeling van der Waals, electrostatic, and desolvation potentials, lending confidence to the predicted binding poses [52,53]. However, we acknowledge that is essential to confirm these interactions and further substantiate the proposed mechanism of action using experimental validation methods, such as surface plasmon resonance (SPR), isothermal titration calorimetry (ITC), or co-crystallization studies [54]. These docking results, consistent with the observed in vitro antiviral activity, highlight the potential of these peptides as broad spectrum antivirals targeting envelope stability while underscoring the importance of future validation efforts.

Targeting the viral envelope is particularly advantageous as it may confer broad-spectrum antiviral activity against a wide range of enveloped viruses. The viral envelope is a critical component of many viruses, playing essential roles in host cell entry, membrane fusion, and viral replication. This makes it a highly conserved and less mutation-prone target compared to viral proteins, which are often subject to genetic variability and the emergence of resistance [55]. In this study, KUNV was selected for in vitro experiments due to its feasibility and compatibility with our experimental framework, as well as its likely relevance as a representative flavivirus model, owing to well-documented structural and biological similarities within the flavivirus group [2,10]. To extend the scope of our findings, molecular docking results were incorporated for ZIKV as an additional flavivirus model. The strong binding observed with ZIKV, coupled with our prior evidence of antiviral activity against Langat virus—a flavivirus closely related to tick-borne encephalitis (TBE) virus—and SARS-CoV-2, suggests that these peptides may exhibit broad spectrum efficacy. This activity appears to extend beyond flaviviruses to include other virus families with similar envelope characteristics, such as coronaviruses, as demonstrated in our previous work on PLNC8 αβ [37]. These findings underscore the potential of viral envelope-targeting peptides as versatile therapeutic agents capable of addressing diverse enveloped viruses.

The potent antiviral activity demonstrated by the novel peptides, especially the truncated versions, paves the way for their further development as therapeutic agents. Future research will focus on several key areas to advance these peptides towards a clinical application. For example, enhancing the stability of these peptides in physiological conditions and developing efficient delivery systems to target infected tissues are critical steps. Moreover, the synergistic effects of PlnEF are noteworthy and warrant further characterization. This study primarily focused on the peptides’ ability to inactivate the virus before cellular entry, aligning with their membrane-disruptive mechanism. However, the potential for post-entry antiviral activity remains an important avenue for exploration. Our previous study has demonstrated the post-infection efficacy of PLNC8 αβ [56], suggesting that similar effects could be observed with PlnEF. Future investigations comparing the post-infection activity of various plantaricins could offer deeper insights into their therapeutic potential. Additionally, it is crucial to evaluate the antiviral efficacy of these peptides in vivo using animal models to understand their pharmacokinetics, toxicity, and therapeutic potential. Moreover, detailed mechanistic studies are needed to fully elucidate the antiviral action of these peptides, which can establish the foundation for the design of even more potent derivatives.

This study highlights the antiviral potential of two-peptide bacteriocins, including PlnEF, PlnJK, and PLNC8 αβ and different plantaricin-derived truncated peptides. Notably, the truncated peptides PLNC8 α1-15 and PLNC8 β1-20 retained significant structural integrity and exhibited robust antiviral activities against KUNV. Integrating in silico predictions with experimental validation was valuable; however, the observed discrepancies between the predicted and observed results emphasize the necessity of empirical testing. Future work will focus on elucidating the precise mechanism of action, peptide stability, delivery systems, and in vivo efficacy studies to further develop these candidates as novel antiviral therapies.

## 4. Materials and Methods

### 4.1. In Silico Prediction of Antiviral Activity

Full-length plantaricins were systematically truncated into 16 amino acid peptides throughout the entire sequence, except for PLNC8 α and PLNC8 β, in which several shorter and longer peptide variants were generated. The general concept includes truncated peptides that cover the entire sequences of the full-length peptides and are composed of amino acids that contribute to at least two α-helix turns. The antiviral activity of full-length and truncated peptides was predicted using the Feature-Informed Reduced Machine Learning for Antiviral Peptide Prediction (FIRM-AVP) [44] and AVPpred [45] software, version 1. These algorithms are used to predict the efficacy of peptides against viruses based on their amino acid sequences. The prediction models incorporate various peptide features, including amino acid composition, physicochemical properties, and sequence motifs, to provide a comprehensive assessment of antiviral potential. All the sequences with predicted antiviral properties were synthesized and tested in vitro against KUNV. FIRM-AVP (Feature-Informed Reduced Model for Antiviral Peptides) is a machine learning-based tool that predicts the antiviral activity of peptides by analyzing primary and secondary structural features. Scores range from 0 to 1, with higher scores indicating a greater likelihood of antiviral activity. A score above 0.5 is generally considered indicative of potential antiviral activity. AVPpred (Antiviral Peptide Prediction Tool) is a computational tool that predicts the antiviral potential of peptides based on their sequence features using machine learning models. Predictions are provided as percentages, with higher percentages indicating a stronger likelihood of antiviral activity. A prediction score above 50% is typically considered indicative of potential antiviral activity.

### 4.2. Peptide Design and Synthesis

PlnA, PlnE, PlnF, PlnJ, and PlnK as well as PLNC8 α, PLNC8 β, and all truncated peptides were synthesized as described earlier [36,57]. The truncated peptides were individually synthesized with free C-terminals (-COOH) and N-terminals (-NH_2_) and not fragmented from their respective full-length counterparts. The sequences of all the peptides can be found in Table 1, Table 2 and Table 3.

### 4.3. In Vitro Antiviral Assays

The antiviral activity of the synthesized peptides was evaluated using plaque reduction assays in Vero cells against West Nile virus Kunjin strain (KUNV, accession number AY274504). Vero cells (Vero E6, ATCC, CRL-1586, Manassas, VA, USA) were maintained in Dulbecco’s Modified Eagle Medium (DMEM, Thermo Fisher Scientific, Gibco, Göteborg, Sweden), supplemented with 10% fetal bovine serum (FBS, Thermo Fisher Scientific, Gibco, Göteborg, Sweden) and antibiotics (PEST, Thermo Fisher Scientific, Gibco, Göteborg, Sweden). For the antiviral assays, Vero cells were seeded in 24-well plates and allowed to reach confluency. The virus suspension (1 × 10^5^ PFU) was pre-incubated with the peptides at 10 µM concentration or lower (in the combination experiment) for 1 h at room temperature. The peptide–virus mixtures were then added to the Vero cell monolayers and incubated for 1 h at 37 °C. Following incubation, the inoculum was removed, and cells were overlaid with Avicel 2.4% overlay media (FMC, Philadelphia, PA, USA). Plates were incubated for 3 days at 37 °C in a CO_2_ incubator. Post-incubation, cells were fixed with 100% methanol and stained with 1% crystal violet to visualize and count viral plaques. The reduction in plaque numbers compared to control wells (virus without peptide) was used to quantify antiviral activity.

### 4.4. Computational Simulations of Peptide Characteristics and Molecular Docking

Characteristics of selected peptides were determined using the RPBS Wed Portal (https://bioserv.rpbs.univ-paris-diderot.fr/index.html, accessed on 25 May 2023). The secondary structures of the peptides were determined by predicting α-helices and coils, hydrophobic cluster analysis, and peptide folding, using the psipred [58], HCA [59], and PEP-FOLD3 [60] tools, respectively. Furthermore, the number of contact neighbors (NCNs), with most interacting residues (MIRs) and least interacting residues (LIRs), was defined based on the thresholds >6 and ≤3, respectively [61].

Molecular docking studies were carried out using FRODOCK [62] to explore the binding interactions between some selected peptides and viral proteins from Zika virus (strain H/PF/2013, PDB: 5IZ7) and West Nile virus (Kunjin strain, PDB: 7KVA). Crystallographic structures of viral proteins were retrieved from the Protein Data Bank (PDB). Prior to docking, protein structures were prepared by removing water molecules, adding hydrogen atoms, and assigning proper charges. An exhaustive docking search was performed for each peptide and receptor using all the potentials (van der Waals, electrostatic, and desolvation).

The docking results were analyzed to identify potential interaction sites and predict binding affinities between peptides and viral proteins. The binding poses with the lowest binding energies were selected for further analysis. Peptide–receptor docked complexes were merged and cleaned using pdb-tools [63] before visualization using PyMol [64]. Insights gained from docking studies were integrated with experimental data on in vitro antiviral activities to elucidate peptide–virus binding mechanisms.

## Figures and Tables

**Table 1:** Amino acid sequences and predicted antiviral activities of plantaricins and truncated peptides. The antiviral activity of full-length and truncated Pln peptides was predicted using FIRM-AVP (*) and AVPpred (#). The antiviral activity of the peptides against the flavivirus KUNV (1 × 10^5^) was determined after exposure to 10 µM in cell culture medium (DMEM) for 1 h. Viral load in Vero cells was quantified by performing the plaque assay. PlnEF and PlnJK were tested for their antiviral activity against KUNV by combining the two individual peptides, e.g., PlnE and PlnF, at a molar ratio of 1:1. NMR structures of all full-length Pln peptides are available in the RCSB protein data bank (https://www.rcsb.org/, accessed on 25 May 2023). Results are presented as the mean with standard deviation of three independent experiments. ND—not determined. PlnA and its derivatives demonstrated moderate predictive and experimentally determined antiviral activity. PlnE and PlnF, both full-length peptides, exhibited modest antiviral activity with variations in their respective predictive scores. PlnJ and PlnK (full-length peptides) and derivatives of PlnJ did not show excessive antiviral activity, despite the high predictive scores. Overall, the results indicate that peptides with predictive scores above the indicative thresholds (FIRM-AVP > 0.5, AVPpred > 50%) generally showed antiviral activity, but discrepancies highlight limitations in the tools. Experimental validation is crucial to address predictive inaccuracies and identify functional truncated peptides.

Peptide	Amino Acid Sequence	FIRM-AVP Score *	AVPpred Score (%) #	KUNV (% Reduction)
PlnA	AYSLQMGATAIKQVKKLFKKWGW	0.97	67.46	27 ± 9
A6	AYSLQMGATAIKQVKKLFKKWGW	0.56	64.93	ND
A7	AYSLQMGATAIKQVKKLFKKWGW	0.67	46.58	ND
A8	AYSLQMGATAIKQVKKLFKKWGW	0.93	43.94	28 ± 8
				
PlnE	FNRGGYNFGKSVRHVVDAIGSVAGIRGILKSIR	0.91	63.51	24 ± 6
				
PlnF	VFHAYSARGVRNNYKSAVGPADWVISAVRGFIHG	0.60	63.90	24 ± 6
F17	VFHAYSARGVRNNYKSAVGPADWVISAVRGFIHG	0.70	28.00	20 ± 5
				
PlnJ	GAWKNFWSSLRKGFYDGEAGRAIRR	0.91	63.59	30 ± 8
J1	GAWKNFWSSLRKGFYDGEAGRAIRR	0.70	64.28	25 ± 8
J2	GAWKNFWSSLRKGFYDGEAGRAIRR	0.64	64.28	ND
J10	GAWKNFWSSLRKGFYDGEAGRAIRR	0.59	30.81	ND
				
PlnK	RRSRKNGIGYAIGYAFGAVERAVLGGSRDYNK	0.30	49.77	18 ± 9

**Table 2:** Amino acid sequences and predicted antiviral activities of truncated peptides derived from PLNC8 α. The antiviral activity of PLNC8 α-derived peptides was predicted using FIRM-AVP (*) and AVPpred (#). All peptide variants were synthesized and tested for their antiviral activity in biological systems against the enveloped flavivirus KUNV (1 × 10^5^) following exposure to 10 µM in the cell culture medium (DMEM) for 1 h. The suspensions were added to Vero cells for 1 h followed by the addition of overlay media and incubation for 3 days. Viral load was quantified by performing the plaque assay. Results are presented as the mean with standard deviation of three independent experiments. The full-length PLNC8 α peptide exhibits moderate antiviral activity with high predictive scores but limited experimental efficacy. Most truncated peptides displayed reduced antiviral activity compared to full-length PLNC8 α, except α1-15, which demonstrated the highest experimental antiviral activity, despite the lower predictive scores. Peptides with extensive C-terminal truncations, such as α23-29 and α16-29, showed minimal activity. Discrepancies were evident between predicted antiviral activity and experimental outcomes, emphasizing the importance of sequence integrity and experimental validation in assessing antiviral potency.

Peptide	Amino Acid Sequence	FIRM-AVP Score *	AVPpred Score (%) #	KUNV (% Reduction)
PLNC8 α	DLTTKLWSSWGYYLGKKARWNLKHPYVQF	0.91	64	35 ± 2
α23-29	DLTTKLWSSWGYYLGKKARWNLKHPYVQF	0.37	35	10 ± 4
α16-29	DLTTKLWSSWGYYLGKKARWNLKHPYVQF	0.23	35	2 ± 0
α9-29	DLTTKLWSSWGYYLGKKARWNLKHPYVQF	0.37	63	10 ± 6
α16-22	DLTTKLWSSWGYYLGKKARWNLKHPYVQF	0.11	34	3 ± 6
α9-22	DLTTKLWSSWGYYLGKKARWNLKHPYVQF	0.06	70	7 ± 7
α1-22	DLTTKLWSSWGYYLGKKARWNLKHPYVQF	0.91	65	28 ± 2
α9-15	DLTTKLWSSWGYYLGKKARWNLKHPYVQF	0.35	49	16 ± 7
α1-15	DLTTKLWSSWGYYLGKKARWNLKHPYVQF	0.04	40	91 ± 2
α1-8	DLTTKLWSSWGYYLGKKARWNLKHPYVQF	0.22	40	20 ± 18

**Table 3:** Amino acid sequences and predicted antiviral activities of truncated and modified peptides derived from PLNC8 β. The antiviral activity of PLNC8 β-derived peptides was predicted using FIRM-AVP (*) and AVPpred (#). The synthesized peptides were tested for their antiviral activity against the flavivirus KUNV (1 × 10^5^) following exposure to 10 µM in the cell culture medium (DMEM) for 1 h. Viral load in Vero cells was quantified by performing the plaque assay. Results are presented as the mean with standard deviation of three independent experiments. KUNV (% reduction) represents the percentage reduction in Kunjin virus (KUNV) load after preincubation with the peptide, as measured by the plaque assay in Vero cells. This value indicates the antiviral efficacy of each peptide. FIRM-AVP (Feature-Informed Reduced Model for Antiviral Peptides). AVPpred (Antiviral Peptide Prediction Tool). The full-length peptide exhibits high antiviral activity, with both predictive scores and experimental results indicating strong efficacy. β1-20 exhibits the highest antiviral activity among truncated peptides, with experimental data surpassing predictive scores. Most truncated peptides showed reduced antiviral activity compared to the full-length PLNC8 β. Extensive C-terminal truncations, such as β28-34 and β21-34, exhibited minimal antiviral activity. A notable discrepancy was observed between predicted antiviral activity and experimental results, as β1-20 demonstrated the highest antiviral efficacy despite lower predictive scores, indicating that experimental factors may influence peptide performance beyond computational predictions.

Peptide	Amino Acid Sequence	FIRM-AVP Score *	AVPpred Score (%) #	KUNV (% Reduction)
PLNC8 β	SVPTSVYTLGIKILWSAYKHRKTIEKSFNKGFYH	0.93	64	70 ± 12
β28-34	SVPTSVYTLGIKILWSAYKHRKTIEKSFNKGFYH	0.37	65	11 ± 15
β21-34	SVPTSVYTLGIKILWSAYKHRKTIEKSFNKGFYH	0.45	63	0 ± 0
β14-34	SVPTSVYTLGIKILWSAYKHRKTIEKSFNKGFYH	0.54	50	5 ± 0.2
β7-34	SVPTSVYTLGIKILWSAYKHRKTIEKSFNKGFYH	0.94	65	52 ± 27
β14-20	SVPTSVYTLGIKILWSAYKHRKTIEKSFNKGFYH	0.33	49	3 ± 4
β7-20	SVPTSVYTLGIKILWSAYKHRKTIEKSFNKGFYH	0.65	45	65 ± 20
β1-20	SVPTSVYTLGIKILWSAYKHRKTIEKSFNKGFYH	0.79	37	92 ± 4
β7-13	SVPTSVYTLGIKILWSAYKHRKTIEKSFNKGFYH	0.29	73	8 ± 4
β(1-6)(7-13)_2_	SVPTSVYTLGIKIYTLGIKILWSAYKHRKTIEKSH	0.38	41	67 ± 9
β1-6	SVPTSVYTLGIKILWSAYKHRKTIEKSFNKGFYH	0.10	6	21 ± 22
β1-13	SVPTSVYTLGIKILWSAYKHRKTIEKSFNKGFYH	0.39	18	5 ± 7
β10-17	SVPTSVYTLGIKILWSAYKHRKTIEKSFNKGFYH	0.55	78	0 ± 0
β4-17	SVPTSVYTLGIKILWSAYKHRKTIEKSFNKGFYH	0.31	33	56 ± 32
β13	SVPTSVYTLGQKILRSARKFGKTIEKSFNKGFYH	0.77	81	8 ± 11
β14	SVPTSVYTLGQKILRSARKFGKDIEKSFNKGFYH	0.72	82	6 ± 8
β15	SVPTSVYTLGIKILRSARKFGKVIEKSFNKGFYH	0.90	84	9 ± 13
β16	SVPTSVYTLGIKILWSARKFGKVIEKSFNKGFYH	0.96	82	84 ± 8
β17	SVPTSVYLLGIKILWSARKFGKVIEKSFNKGFYH	0.95	80	14 ± 12
β18	SVPTSVYLLGIKILWKARKFGKVIEKSFNKGFYH	0.98	85	12 ± 1

**Table 4:** Absolute energy score of the peptides docking with their respective receptors. Predicted interactions of PLNC8α and PLNC8β and their truncated variants (PLNC8α1-15 and PLNC8β1-20) with the structural E protein of Zika and Kunjin were performed using FRODOCK. An exhaustive docking search was conducted for each peptide and receptor using all the potentials (van der Waals, electrostatic, and desolvation).

Target	PDB ID	PLNC8α	PLNC8α1-15	PLNC8β	PLNC8β1-20
Zika virus	S1Z7	5210	4541	5602	4948
WNV, Kunjin	7KVA	5541	3877	6183	5308

## Data Availability

The original contributions presented in the study are included in the article; further inquiries can be directed to the corresponding author.
